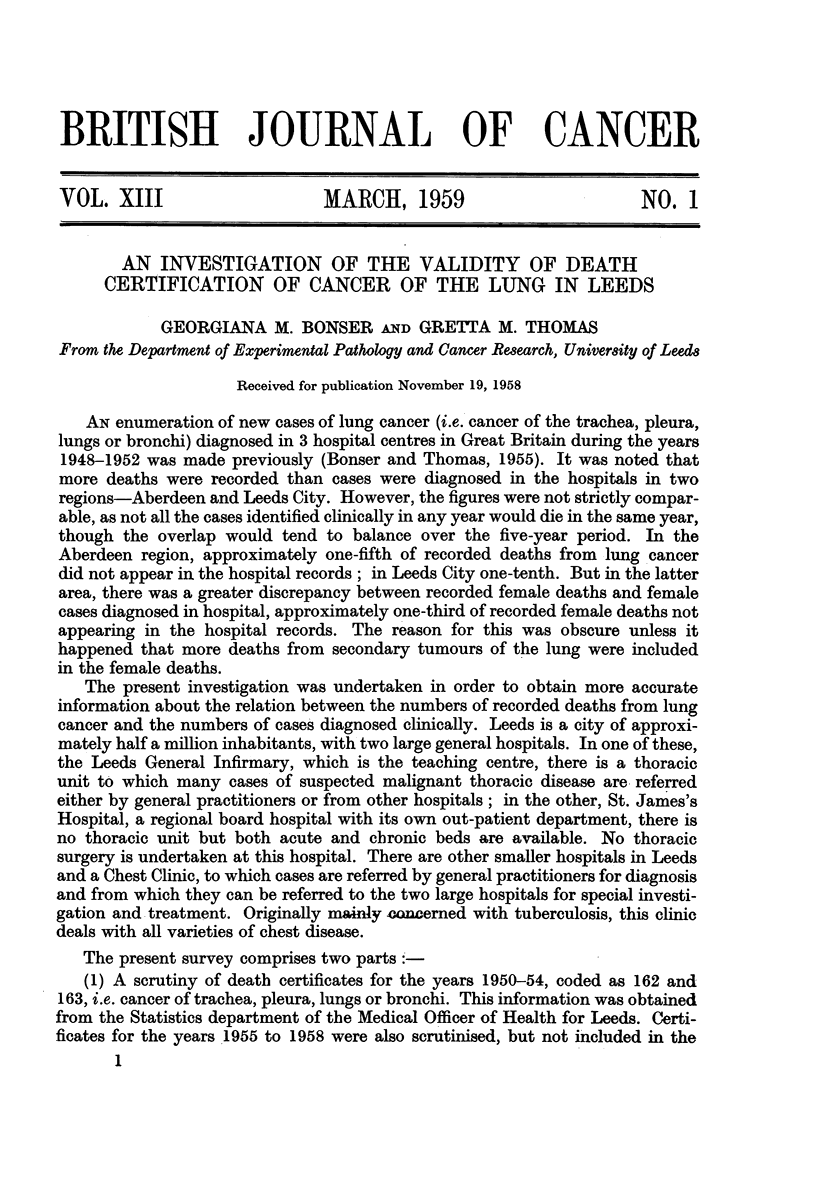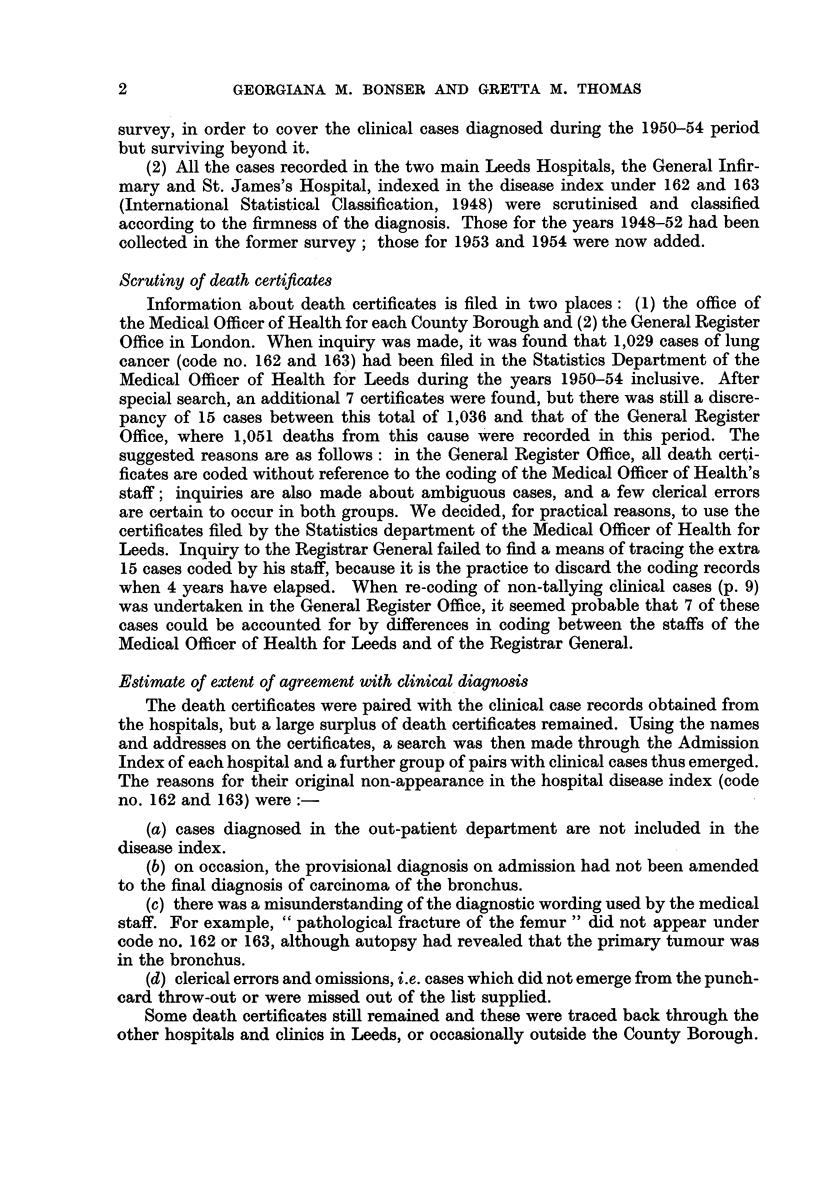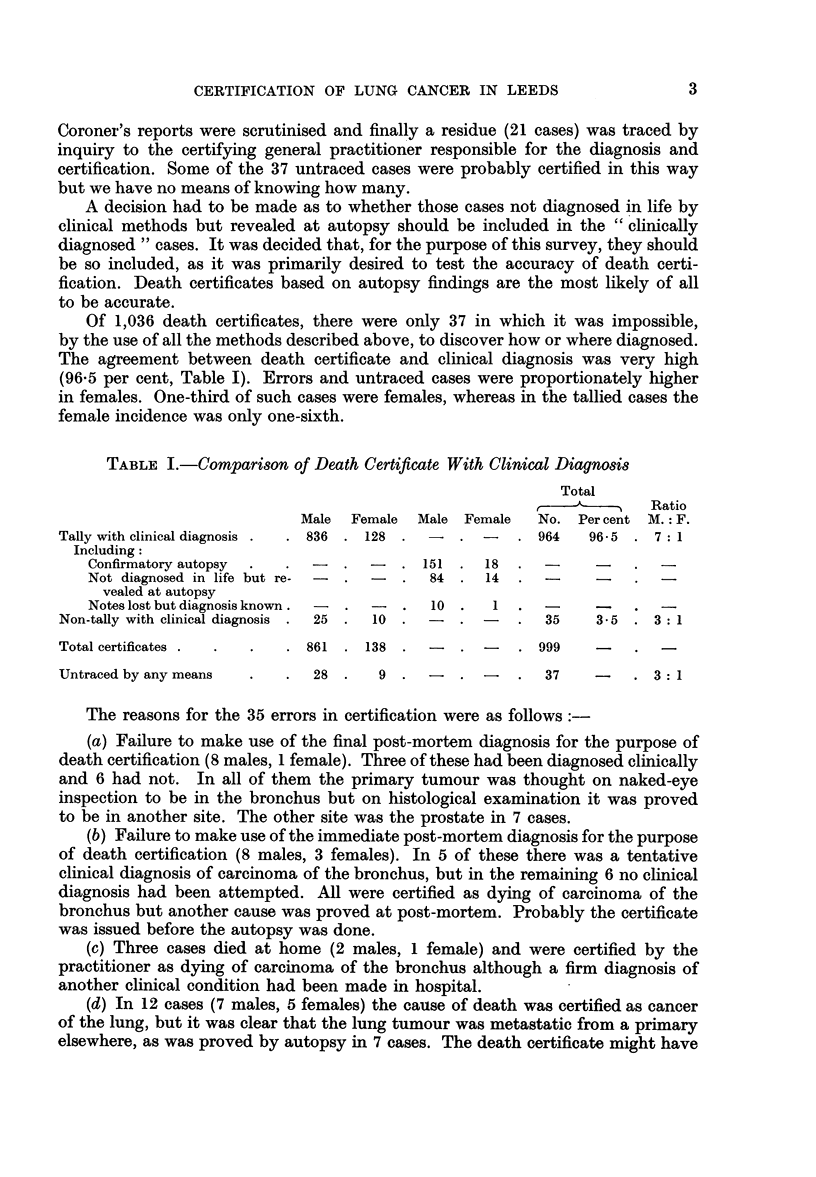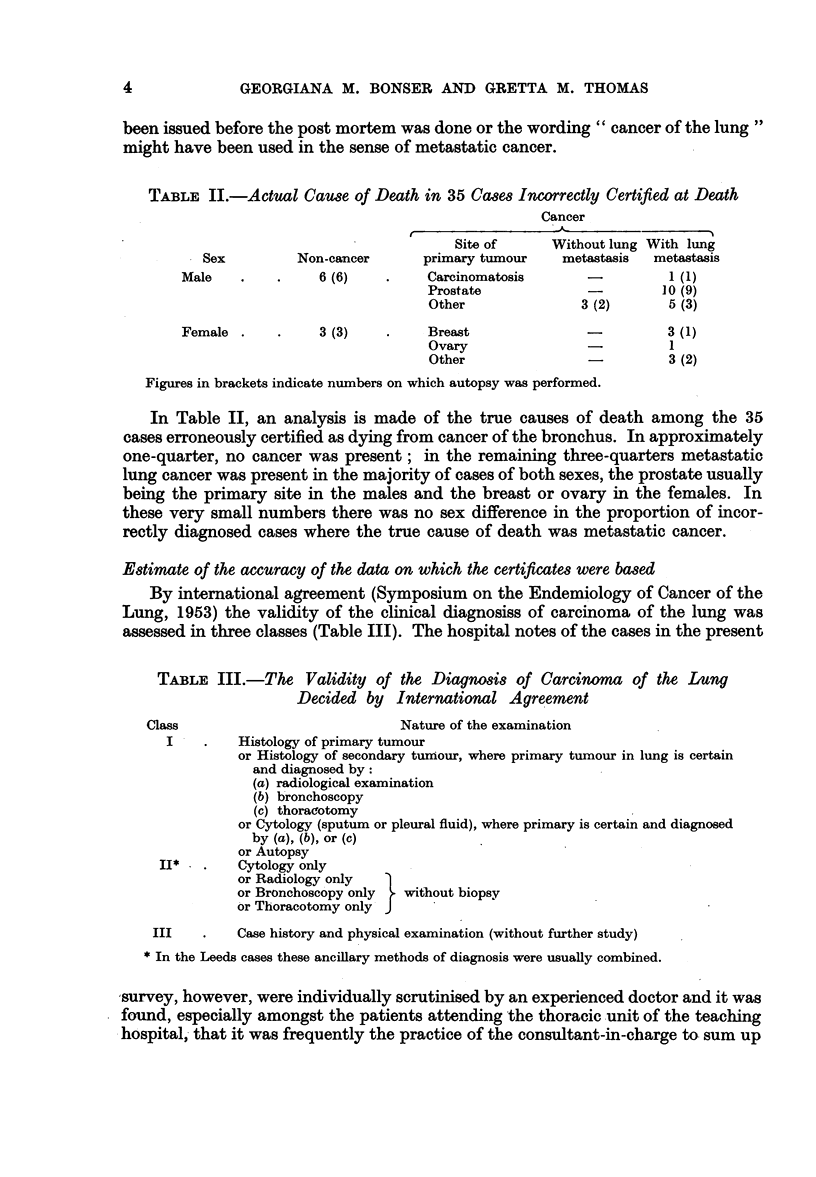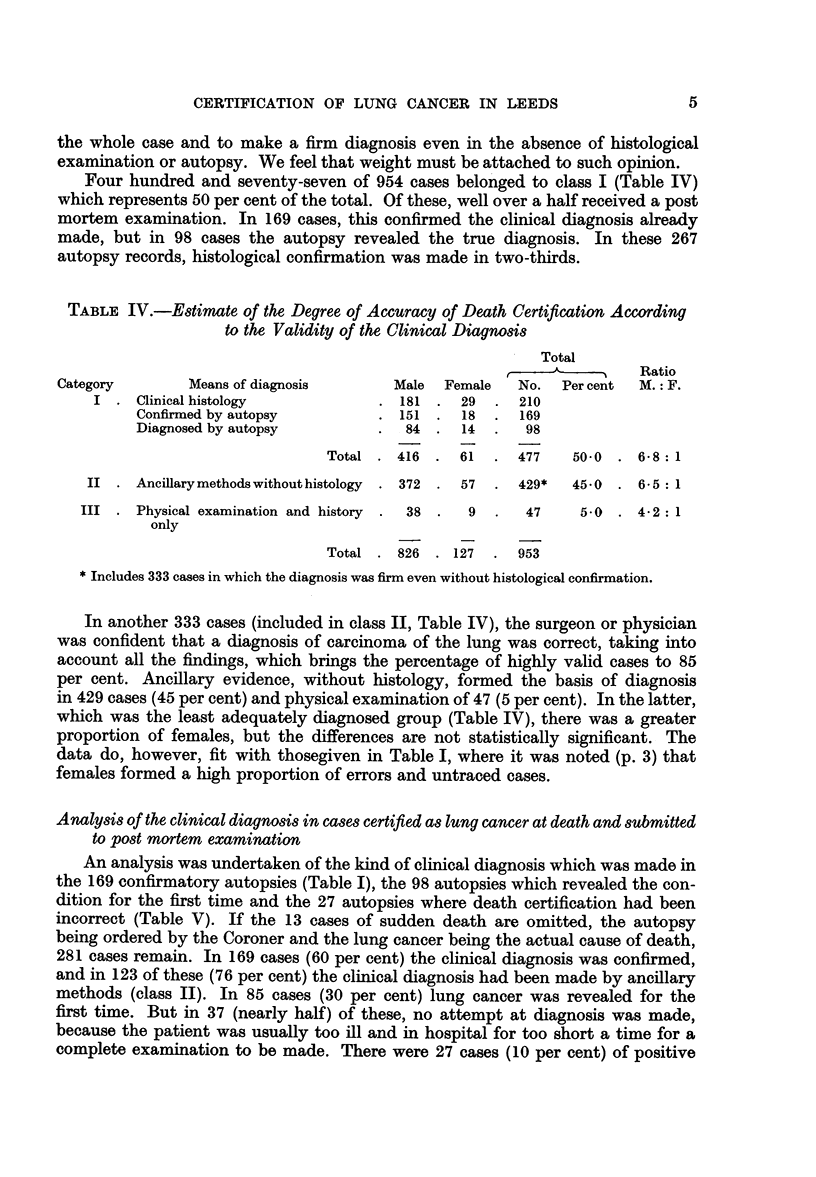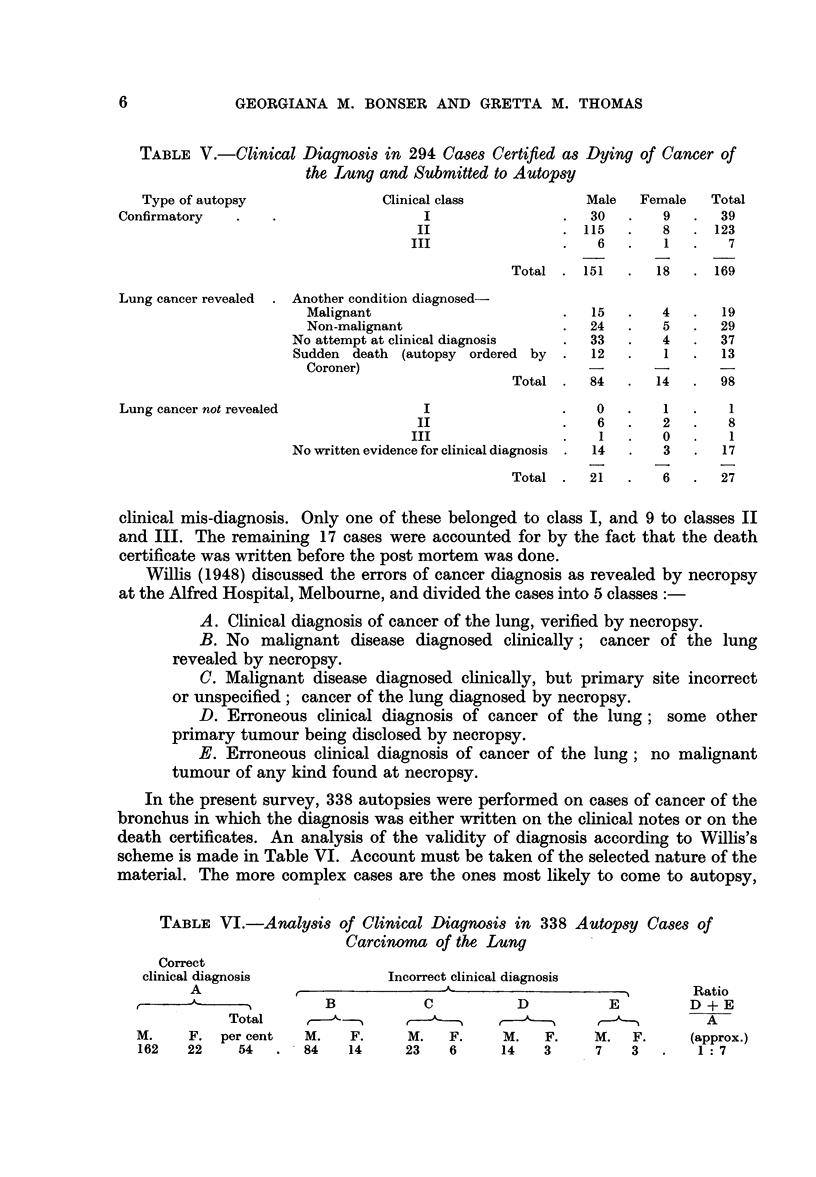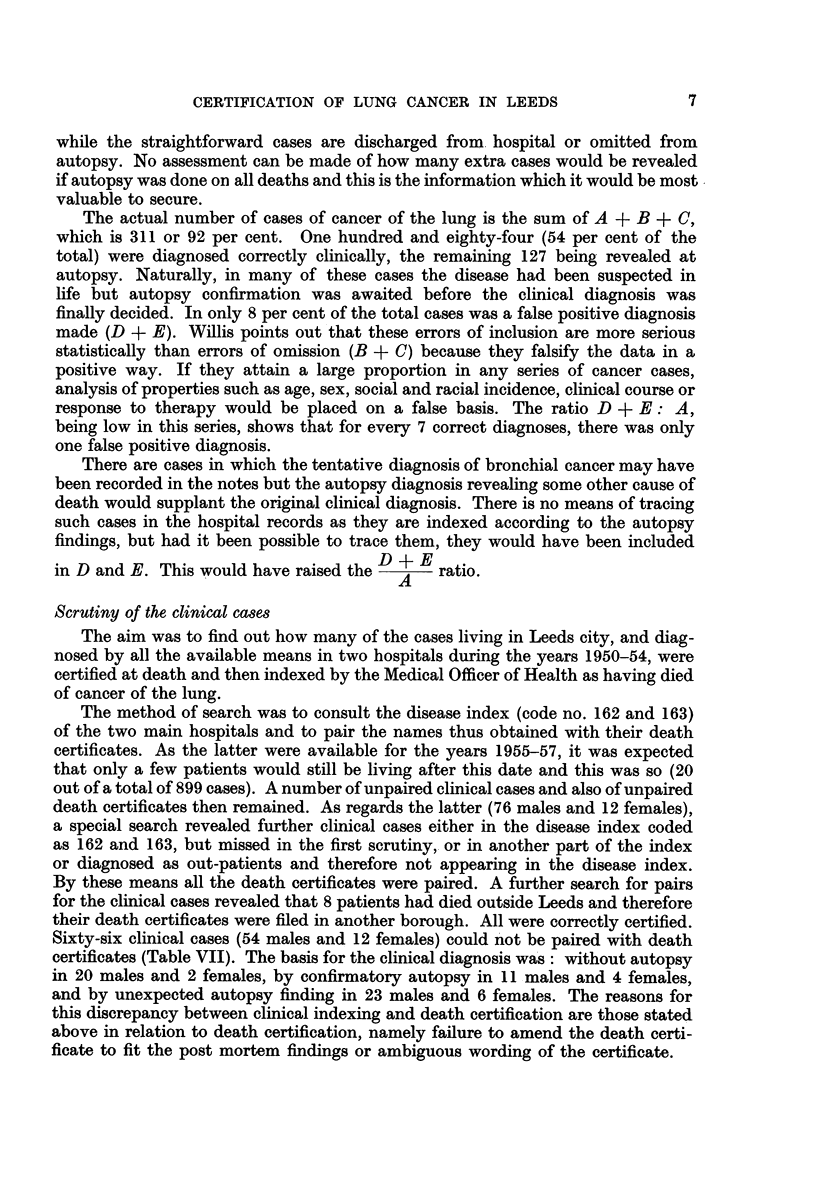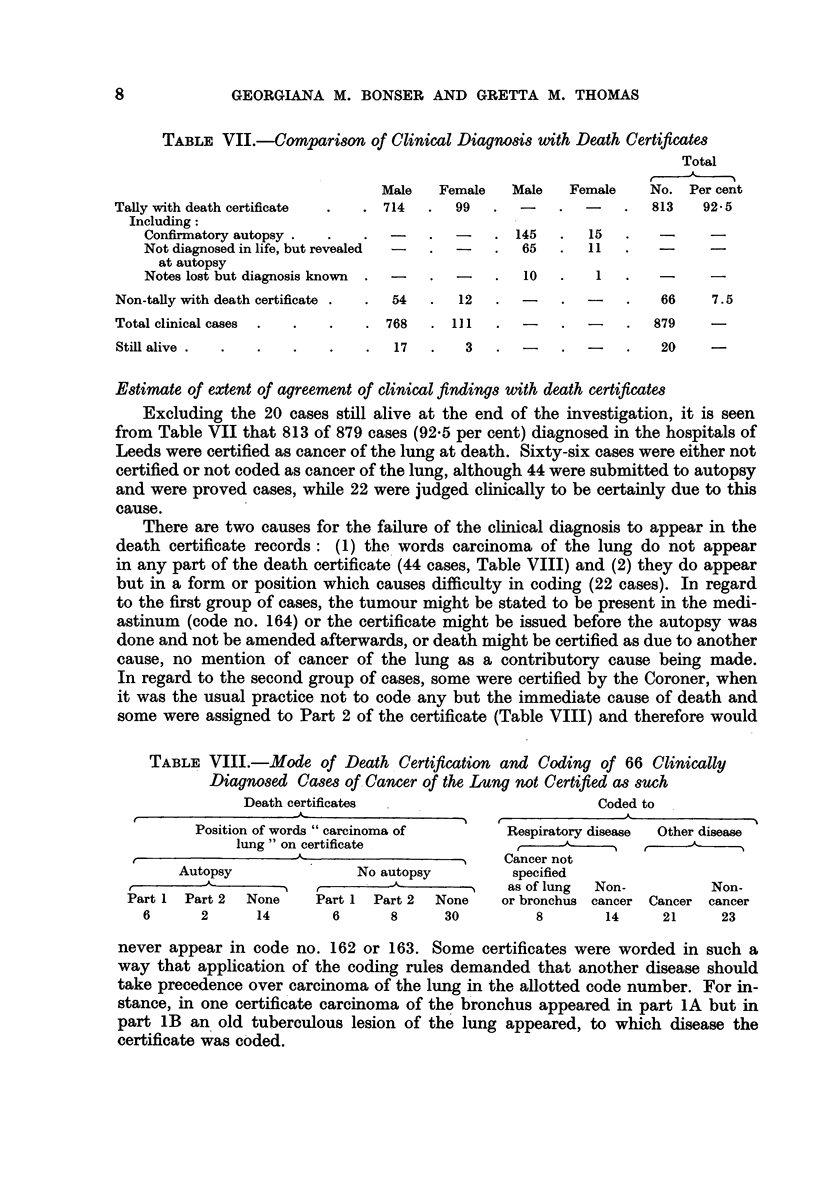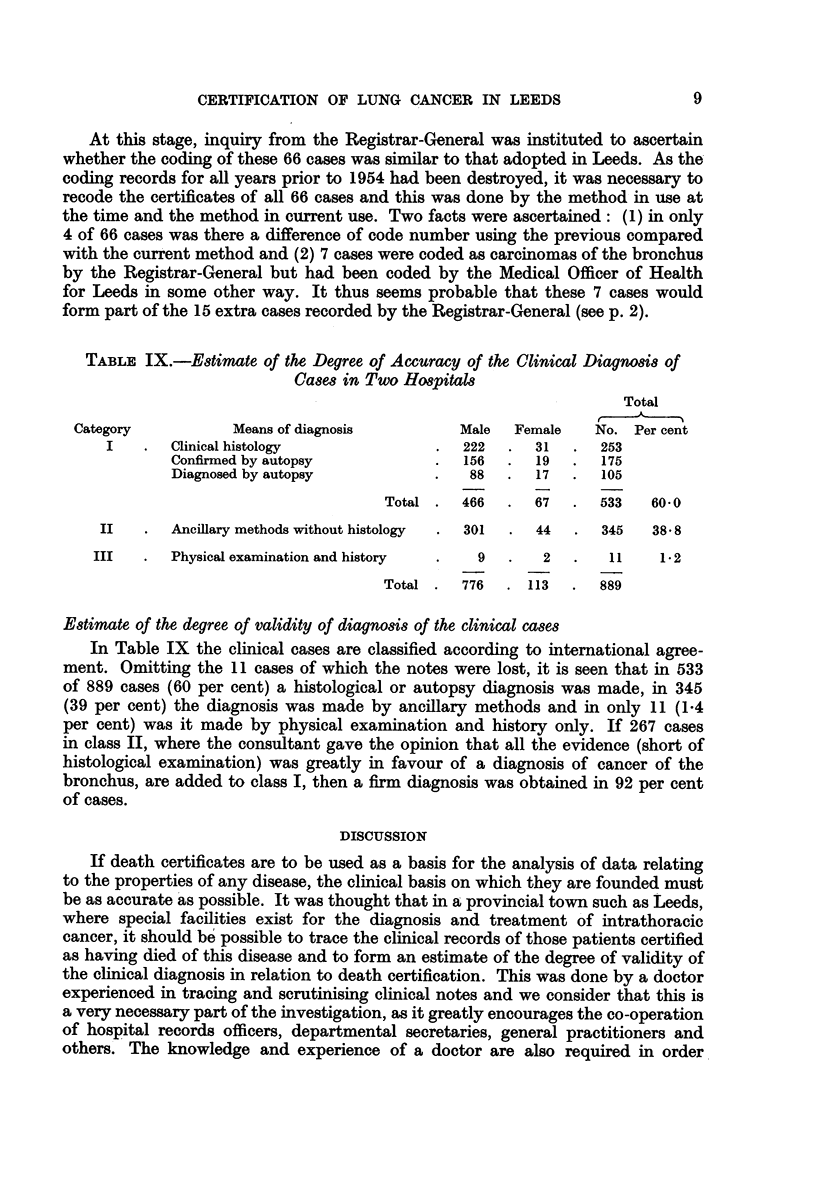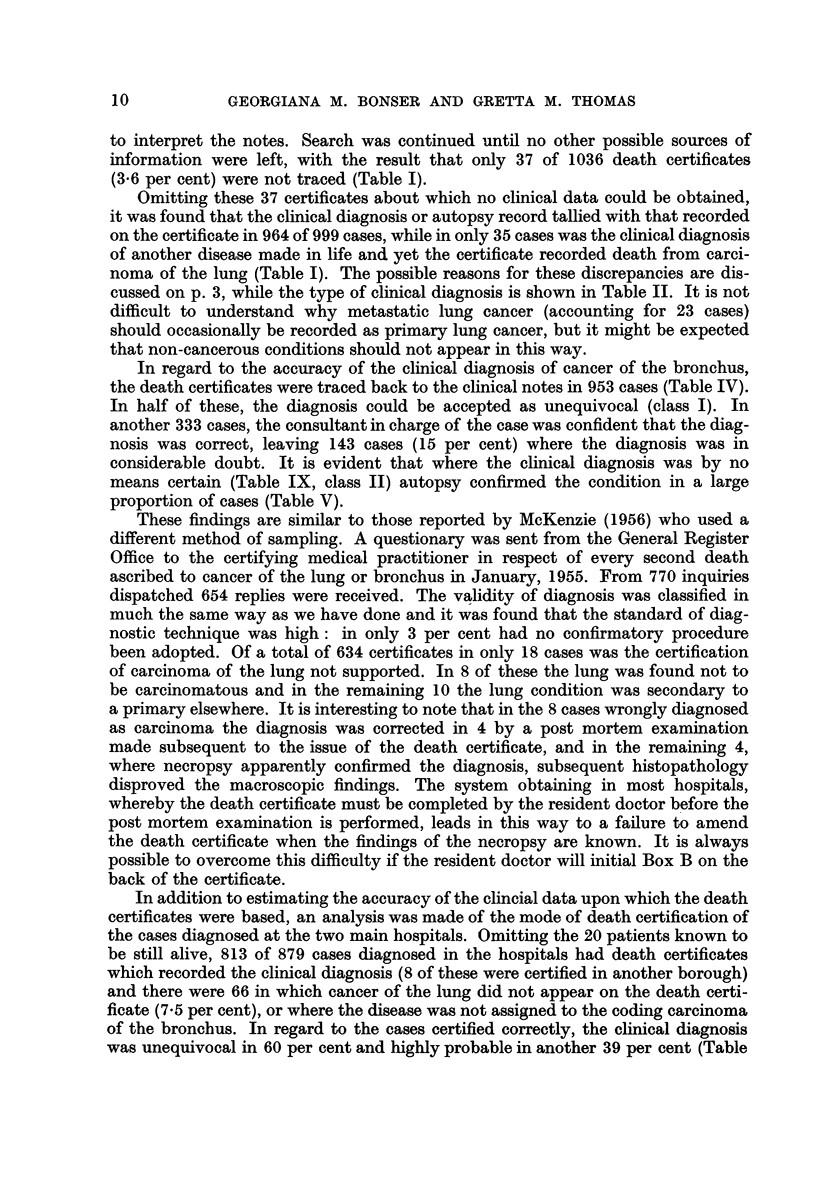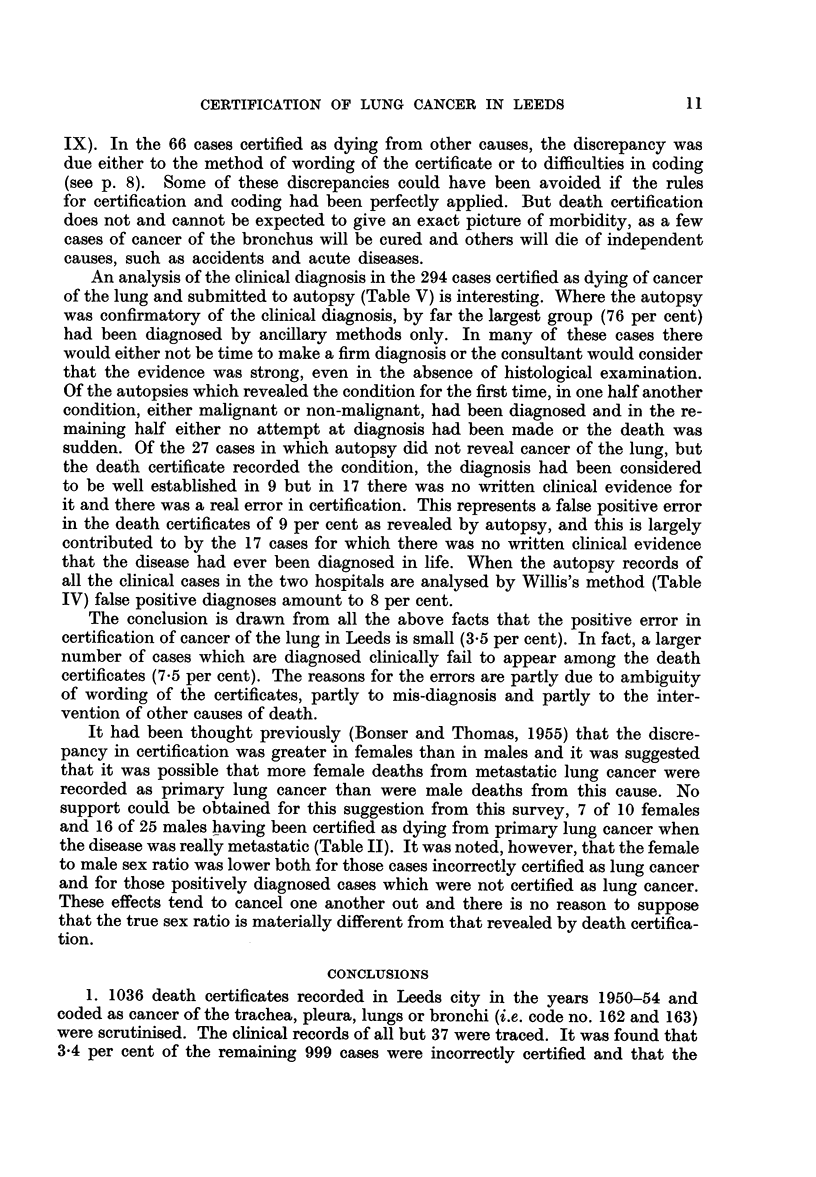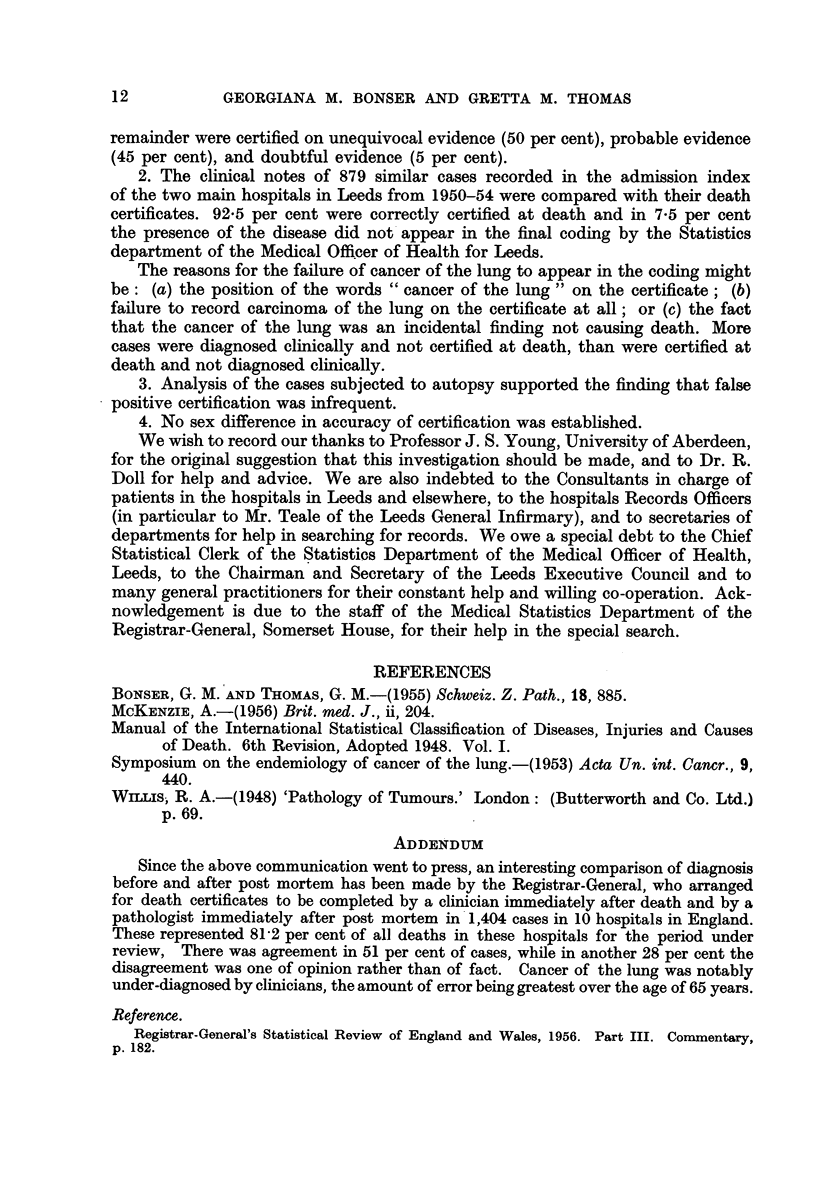# An Investigation of the Validity of Death Certification of Cancer of the Lung in Leeds

**DOI:** 10.1038/bjc.1959.1

**Published:** 1959-03

**Authors:** Georgiana M. Bonser, Gretta M. Thomas


BRITISH JOURNAL OF CANCER

VOL. XIII MARCH, 1959 NO. 1

### AN INVESTIGATION OF THE VALIDITY OF DEATH CERTIFICATION OF CANCER OF THE LUNG IN LEEDS

GEORGIANA M. BONSER AND GRETTA M. THOMAS

*From the Department of Experimental Pathology and Cancer Research, University of Leeds*

Received for publication November 19, 1958

An enumeration of new cases of lung cancer (*i.e.* cancer of the trachea, pleura, lungs or bronchi) diagnosed in 3 hospital centres in Great Britain during the years 1948–1952 was made previously (Bonser and Thomas, 1955). It was noted that more deaths were recorded than cases were diagnosed in the hospitals in two regions—Aberdeen and Leeds City. However, the figures were not strictly comparable, as not all the cases identified clinically in any year would die in the same year, though the overlap would tend to balance over the five-year period. In the Aberdeen region, approximately one-fifth of recorded deaths from lung cancer did not appear in the hospital records ; in Leeds City one-tenth. But in the latter area, there was a greater discrepancy between recorded female deaths and female cases diagnosed in hospital, approximately one-third of recorded female deaths not appearing in the hospital records. The reason for this was obscure unless it happened that more deaths from secondary tumours of the lung were included in the female deaths.

The present investigation was undertaken in order to obtain more accurate information about the relation between the numbers of recorded deaths from lung cancer and the numbers of cases diagnosed clinically. Leeds is a city of approximately half a million inhabitants, with two large general hospitals. In one of these, the Leeds General Infirmary, which is the teaching centre, there is a thoracic unit to which many cases of suspected malignant thoracic disease are referred either by general practitioners or from other hospitals ; in the other, St. James's Hospital, a regional board hospital with its own out-patient department, there is no thoracic unit but both acute and chronic beds are available. No thoracic surgery is undertaken at this hospital. There are other smaller hospitals in Leeds and a Chest Clinic, to which cases are referred by general practitioners for diagnosis and from which they can be referred to the two large hospitals for special investigation and treatment. Originally mainly concerned with tuberculosis, this clinic deals with all varieties of chest disease.

The present survey comprises two parts :—

(1) A scrutiny of death certificates for the years 1950–54, coded as 162 and 163, *i.e.* cancer of trachea, pleura, lungs or bronchi. This information was obtained from the Statistics department of the Medical Officer of Health for Leeds. Certificates for the years 1955 to 1958 were also scrutinised, but not included in the survey, in order to cover the clinical cases diagnosed during the 1950–54 period but surviving beyond it.

(2) All the cases recorded in the two main Leeds Hospitals, the General Infirmary and St. James's Hospital, indexed in the disease index under 162 and 163 (International Statistical Classification, 1948) were scrutinised and classified according to the firmness of the diagnosis. Those for the years 1948–52 had been collected in the former survey ; those for 1953 and 1954 were now added.

*Scrutiny of death certificates*

Information about death certificates is filed in two places : (1) the office of the Medical Officer of Health for each County Borough and (2) the General Register Office in London. When inquiry was made, it was found that 1,029 cases of lung cancer (code no. 162 and 163) had been filed in the Statistics Department of the Medical Officer of Health for Leeds during the years 1950–54 inclusive. After special search, an additional 7 certificates were found, but there was still a discrepancy of 15 cases between this total of 1,036 and that of the General Register Office, where 1,051 deaths from this cause were recorded in this period. The suggested reasons are as follows : in the General Register Office, all death certificates are coded without reference to the coding of the Medical Officer of Health's staff ; inquiries are also made about ambiguous cases, and a few clerical errors are certain to occur in both groups. We decided, for practical reasons, to use the certificates filed by the Statistics department of the Medical Officer of Health for Leeds. Inquiry to the Registrar General failed to find a means of tracing the extra 15 cases coded by his staff, because it is the practice to discard the coding records when 4 years have elapsed. When re-coding of non-tallying clinical cases (p. 9) was undertaken in the General Register Office, it seemed probable that 7 of these cases could be accounted for by differences in coding between the staffs of the Medical Officer of Health for Leeds and of the Registrar General.

*Estimate of extent of agreement with clinical diagnosis*

The death certificates were paired with the clinical case records obtained from the hospitals, but a large surplus of death certificates remained. Using the names and addresses on the certificates, a search was then made through the Admission Index of each hospital and a further group of pairs with clinical cases thus emerged. The reasons for their original non-appearance in the hospital disease index (code no. 162 and 163) were :—

(*a*) cases diagnosed in the out-patient department are not included in the disease index.

(*b*) on occasion, the provisional diagnosis on admission had not been amended to the final diagnosis of carcinoma of the bronchus.

(*c*) there was a misunderstanding of the diagnostic wording used by the medical staff. For example, " pathological fracture of the femur " did not appear under code no. 162 or 163, although autopsy had revealed that the primary tumour was in the bronchus.

(*d*) clerical errors and omissions, *i.e.* cases which did not emerge from the punch-card throw-out or were missed out of the list supplied.

Some death certificates still remained and these were traced back through the other hospitals and clinics in Leeds, or occasionally outside the County Borough.

Coroner's reports were scrutinised and finally a residue (21 cases) was traced by inquiry to the certifying general practitioner responsible for the diagnosis and certification. Some of the 37 untraced cases were probably certified in this way but we have no means of knowing how many.

A decision had to be made as to whether those cases not diagnosed in life by clinical methods but revealed at autopsy should be included in the " clinically diagnosed " cases. It was decided that, for the purpose of this survey, they should be so included, as it was primarily desired to test the accuracy of death certification. Death certificates based on autopsy findings are the most likely of all to be accurate.

Of 1,036 death certificates, there were only 37 in which it was impossible, by the use of all the methods described above, to discover how or where diagnosed. The agreement between death certificate and clinical diagnosis was very high (96·5 per cent, Table I). Errors and untraced cases were proportionately higher in females. One-third of such cases were females, whereas in the tallied cases the female incidence was only one-sixth.

TABLE I.—*Comparison of Death Certificate With Clinical Diagnosis*

| | Male | Female | Male | Female | Total | | Ratio |
|---|---|---|---|---|---|---|---|
| | | | | | No. | Per cent | M. : F. |
| Tally with clinical diagnosis | 836 | 128 | — | — | 964 | 96·5 | 7 : 1 |
| Including : | | | | | | | |
| Confirmatory autopsy | — | — | 151 | 18 | — | — | — |
| Not diagnosed in life but revealed at autopsy | — | — | 84 | 14 | — | — | — |
| Notes lost but diagnosis known | — | — | 10 | 1 | — | — | — |
| Non-tally with clinical diagnosis | 25 | 10 | — | — | 35 | 3·5 | 3 : 1 |
| Total certificates | 861 | 138 | — | — | 999 | — | — |
| Untraced by any means | 28 | 9 | — | — | 37 | — | 3 : 1 |

The reasons for the 35 errors in certification were as follows :—

(*a*) Failure to make use of the final post-mortem diagnosis for the purpose of death certification (8 males, 1 female). Three of these had been diagnosed clinically and 6 had not. In all of them the primary tumour was thought on naked-eye inspection to be in the bronchus but on histological examination it was proved to be in another site. The other site was the prostate in 7 cases.

(*b*) Failure to make use of the immediate post-mortem diagnosis for the purpose of death certification (8 males, 3 females). In 5 of these there was a tentative clinical diagnosis of carcinoma of the bronchus, but in the remaining 6 no clinical diagnosis had been attempted. All were certified as dying of carcinoma of the bronchus but another cause was proved at post-mortem. Probably the certificate was issued before the autopsy was done.

(*c*) Three cases died at home (2 males, 1 female) and were certified by the practitioner as dying of carcinoma of the bronchus although a firm diagnosis of another clinical condition had been made in hospital.

(*d*) In 12 cases (7 males, 5 females) the cause of death was certified as cancer of the lung, but it was clear that the lung tumour was metastatic from a primary elsewhere, as was proved by autopsy in 7 cases. The death certificate might have been issued before the post mortem was done or the wording " cancer of the lung " might have been used in the sense of metastatic cancer.

TABLE II.—*Actual Cause of Death in 35 Cases Incorrectly Certified at Death*

| | | Cancer | | |
|---|---|---|---|---|
| Sex | Non-cancer | Site of primary tumour | Without lung metastasis | With lung metastasis |
| Male | 6 (6) | Carcinomatosis | — | 1 (1) |
| | | Prostate | — | 10 (9) |
| | | Other | 3 (2) | 5 (3) |
| Female | 3 (3) | Breast | — | 3 (1) |
| | | Ovary | — | 1 |
| | | Other | — | 3 (2) |

Figures in brackets indicate numbers on which autopsy was performed.

In Table II, an analysis is made of the true causes of death among the 35 cases erroneously certified as dying from cancer of the bronchus. In approximately one-quarter, no cancer was present ; in the remaining three-quarters metastatic lung cancer was present in the majority of cases of both sexes, the prostate usually being the primary site in the males and the breast or ovary in the females. In these very small numbers there was no sex difference in the proportion of incorrectly diagnosed cases where the true cause of death was metastatic cancer.

*Estimate of the accuracy of the data on which the certificates were based*

By international agreement (Symposium on the Endemiology of Cancer of the Lung, 1953) the validity of the clinical diagnosiss of carcinoma of the lung was assessed in three classes (Table III). The hospital notes of the cases in the present

TABLE III.—*The Validity of the Diagnosis of Carcinoma of the Lung Decided by International Agreement*

| Class | Nature of the examination |
|---|---|
| I | Histology of primary tumour<br>or Histology of secondary tumour, where primary tumour in lung is certain and diagnosed by :<br>(*a*) radiological examination<br>(*b*) bronchoscopy<br>(*c*) thoracotomy<br>or Cytology (sputum or pleural fluid), where primary is certain and diagnosed by (*a*), (*b*), or (*c*)<br>or Autopsy |
| II* | Cytology only<br>or Radiology only<br>or Bronchoscopy only<br>or Thoracotomy only } without biopsy |
| III | Case history and physical examination (without further study) |

* In the Leeds cases these ancillary methods of diagnosis were usually combined.

survey, however, were individually scrutinised by an experienced doctor and it was found, especially amongst the patients attending the thoracic unit of the teaching hospital, that it was frequently the practice of the consultant-in-charge to sum up the whole case and to make a firm diagnosis even in the absence of histological examination or autopsy. We feel that weight must be attached to such opinion.

Four hundred and seventy-seven of 954 cases belonged to class I (Table IV) which represents 50 per cent of the total. Of these, well over a half received a post mortem examination. In 169 cases, this confirmed the clinical diagnosis already made, but in 98 cases the autopsy revealed the true diagnosis. In these 267 autopsy records, histological confirmation was made in two-thirds.

TABLE IV.—*Estimate of the Degree of Accuracy of Death Certification According to the Validity of the Clinical Diagnosis*

| Category | Means of diagnosis | Male | Female | Total No. | Total Per cent | Ratio M. : F. |
|---|---|---|---|---|---|---|
| I | Clinical histology | 181 | 29 | 210 | | |
| | Confirmed by autopsy | 151 | 18 | 169 | | |
| | Diagnosed by autopsy | 84 | 14 | 98 | | |
| | Total | 416 | 61 | 477 | 50·0 | 6·8 : 1 |
| II | Ancillary methods without histology | 372 | 57 | 429* | 45·0 | 6·5 : 1 |
| III | Physical examination and history only | 38 | 9 | 47 | 5·0 | 4·2 : 1 |
| | Total | 826 | 127 | 953 | | |

* Includes 333 cases in which the diagnosis was firm even without histological confirmation.

In another 333 cases (included in class II, Table IV), the surgeon or physician was confident that a diagnosis of carcinoma of the lung was correct, taking into account all the findings, which brings the percentage of highly valid cases to 85 per cent. Ancillary evidence, without histology, formed the basis of diagnosis in 429 cases (45 per cent) and physical examination of 47 (5 per cent). In the latter, which was the least adequately diagnosed group (Table IV), there was a greater proportion of females, but the differences are not statistically significant. The data do, however, fit with thosegiven in Table I, where it was noted (p. 3) that females formed a high proportion of errors and untraced cases.

*Analysis of the clinical diagnosis in cases certified as lung cancer at death and submitted to post mortem examination*

An analysis was undertaken of the kind of clinical diagnosis which was made in the 169 confirmatory autopsies (Table I), the 98 autopsies which revealed the condition for the first time and the 27 autopsies where death certification had been incorrect (Table V). If the 13 cases of sudden death are omitted, the autopsy being ordered by the Coroner and the lung cancer being the actual cause of death, 281 cases remain. In 169 cases (60 per cent) the clinical diagnosis was confirmed, and in 123 of these (76 per cent) the clinical diagnosis had been made by ancillary methods (class II). In 85 cases (30 per cent) lung cancer was revealed for the first time. But in 37 (nearly half) of these, no attempt at diagnosis was made, because the patient was usually too ill and in hospital for too short a time for a complete examination to be made. There were 27 cases (10 per cent) of positive

TABLE V.—*Clinical Diagnosis in 294 Cases Certified as Dying of Cancer of the Lung and Submitted to Autopsy*

| Type of autopsy | Clinical class | Male | Female | Total |
|---|---|---|---|---|
| Confirmatory | I | 30 | 9 | 39 |
| | II | 115 | 8 | 123 |
| | III | 6 | 1 | 7 |
| | Total | 151 | 18 | 169 |
| Lung cancer revealed | Another condition diagnosed— | | | |
| | Malignant | 15 | 4 | 19 |
| | Non-malignant | 24 | 5 | 29 |
| | No attempt at clinical diagnosis | 33 | 4 | 37 |
| | Sudden death (autopsy ordered by Coroner) | 12 | 1 | 13 |
| | Total | 84 | 14 | 98 |
| Lung cancer *not* revealed | I | 0 | 1 | 1 |
| | II | 6 | 2 | 8 |
| | III | 1 | 0 | 1 |
| | No written evidence for clinical diagnosis | 14 | 3 | 17 |
| | Total | 21 | 6 | 27 |

clinical mis-diagnosis. Only one of these belonged to class I, and 9 to classes II and III. The remaining 17 cases were accounted for by the fact that the death certificate was written before the post mortem was done.

Willis (1948) discussed the errors of cancer diagnosis as revealed by necropsy at the Alfred Hospital, Melbourne, and divided the cases into 5 classes :—

*A*. Clinical diagnosis of cancer of the lung, verified by necropsy.

*B*. No malignant disease diagnosed clinically ; cancer of the lung revealed by necropsy.

*C*. Malignant disease diagnosed clinically, but primary site incorrect or unspecified ; cancer of the lung diagnosed by necropsy.

*D*. Erroneous clinical diagnosis of cancer of the lung ; some other primary tumour being disclosed by necropsy.

*E*. Erroneous clinical diagnosis of cancer of the lung ; no malignant tumour of any kind found at necropsy.

In the present survey, 338 autopsies were performed on cases of cancer of the bronchus in which the diagnosis was either written on the clinical notes or on the death certificates. An analysis of the validity of diagnosis according to Willis's scheme is made in Table VI. Account must be taken of the selected nature of the material. The more complex cases are the ones most likely to come to autopsy,

TABLE VI.—*Analysis of Clinical Diagnosis in 338 Autopsy Cases of Carcinoma of the Lung*

| Correct clinical diagnosis A | | | Incorrect clinical diagnosis | | | | | | | | Ratio $\frac{D+E}{A}$ |
|---|---|---|---|---|---|---|---|---|---|---|---|
| | | | B | | C | | D | | E | | |
| M. | F. | Total per cent | M. | F. | M. | F. | M. | F. | M. | F. | (approx.) |
| 162 | 22 | 54 | 84 | 14 | 23 | 6 | 14 | 3 | 7 | 3 | 1 : 7 |

while the straightforward cases are discharged from hospital or omitted from autopsy. No assessment can be made of how many extra cases would be revealed if autopsy was done on all deaths and this is the information which it would be most valuable to secure.

The actual number of cases of cancer of the lung is the sum of $A + B + C$, which is 311 or 92 per cent. One hundred and eighty-four (54 per cent of the total) were diagnosed correctly clinically, the remaining 127 being revealed at autopsy. Naturally, in many of these cases the disease had been suspected in life but autopsy confirmation was awaited before the clinical diagnosis was finally decided. In only 8 per cent of the total cases was a false positive diagnosis made ($D + E$). Willis points out that these errors of inclusion are more serious statistically than errors of omission ($B + C$) because they falsify the data in a positive way. If they attain a large proportion in any series of cancer cases, analysis of properties such as age, sex, social and racial incidence, clinical course or response to therapy would be placed on a false basis. The ratio $D + E : A$, being low in this series, shows that for every 7 correct diagnoses, there was only one false positive diagnosis.

There are cases in which the tentative diagnosis of bronchial cancer may have been recorded in the notes but the autopsy diagnosis revealing some other cause of death would supplant the original clinical diagnosis. There is no means of tracing such cases in the hospital records as they are indexed according to the autopsy findings, but had it been possible to trace them, they would have been included in $D$ and $E$. This would have raised the $\frac{D + E}{A}$ ratio.

*Scrutiny of the clinical cases*

The aim was to find out how many of the cases living in Leeds city, and diagnosed by all the available means in two hospitals during the years 1950–54, were certified at death and then indexed by the Medical Officer of Health as having died of cancer of the lung.

The method of search was to consult the disease index (code no. 162 and 163) of the two main hospitals and to pair the names thus obtained with their death certificates. As the latter were available for the years 1955–57, it was expected that only a few patients would still be living after this date and this was so (20 out of a total of 899 cases). A number of unpaired clinical cases and also of unpaired death certificates then remained. As regards the latter (76 males and 12 females), a special search revealed further clinical cases either in the disease index coded as 162 and 163, but missed in the first scrutiny, or in another part of the index or diagnosed as out-patients and therefore not appearing in the disease index. By these means all the death certificates were paired. A further search for pairs for the clinical cases revealed that 8 patients had died outside Leeds and therefore their death certificates were filed in another borough. All were correctly certified. Sixty-six clinical cases (54 males and 12 females) could not be paired with death certificates (Table VII). The basis for the clinical diagnosis was : without autopsy in 20 males and 2 females, by confirmatory autopsy in 11 males and 4 females, and by unexpected autopsy finding in 23 males and 6 females. The reasons for this discrepancy between clinical indexing and death certification are those stated above in relation to death certification, namely failure to amend the death certificate to fit the post mortem findings or ambiguous wording of the certificate.

TABLE VII.—*Comparison of Clinical Diagnosis with Death Certificates*

| | Male | Female | Male | Female | Total | |
|---|---|---|---|---|---|---|
| | | | | | No. | Per cent |
| Tally with death certificate | 714 | 99 | — | — | 813 | 92·5 |
| Including : | | | | | | |
| Confirmatory autopsy | — | — | 145 | 15 | — | — |
| Not diagnosed in life, but revealed at autopsy | — | — | 65 | 11 | — | — |
| Notes lost but diagnosis known | — | — | 10 | 1 | — | — |
| Non-tally with death certificate | 54 | 12 | — | — | 66 | 7.5 |
| Total clinical cases | 768 | 111 | — | — | 879 | — |
| Still alive | 17 | 3 | — | — | 20 | — |

*Estimate of extent of agreement of clinical findings with death certificates*

Excluding the 20 cases still alive at the end of the investigation, it is seen from Table VII that 813 of 879 cases (92·5 per cent) diagnosed in the hospitals of Leeds were certified as cancer of the lung at death. Sixty-six cases were either not certified or not coded as cancer of the lung, although 44 were submitted to autopsy and were proved cases, while 22 were judged clinically to be certainly due to this cause.

There are two causes for the failure of the clinical diagnosis to appear in the death certificate records : (1) the words carcinoma of the lung do not appear in any part of the death certificate (44 cases, Table VIII) and (2) they do appear but in a form or position which causes difficulty in coding (22 cases). In regard to the first group of cases, the tumour might be stated to be present in the mediastinum (code no. 164) or the certificate might be issued before the autopsy was done and not be amended afterwards, or death might be certified as due to another cause, no mention of cancer of the lung as a contributory cause being made. In regard to the second group of cases, some were certified by the Coroner, when it was the usual practice not to code any but the immediate cause of death and some were assigned to Part 2 of the certificate (Table VIII) and therefore would

TABLE VIII.—*Mode of Death Certification and Coding of* 66 *Clinically Diagnosed Cases of Cancer of the Lung not Certified as such*

| Death certificates | | | | | | Coded to | | | |
|---|---|---|---|---|---|---|---|---|---|
| Position of words " carcinoma of lung " on certificate | | | | | | Respiratory disease | | Other disease | |
| Autopsy | | | No autopsy | | | | | | |
| Part 1 | Part 2 | None | Part 1 | Part 2 | None | Cancer not specified as of lung or bronchus | Non-cancer | Cancer | Non-cancer |
| 6 | 2 | 14 | 6 | 8 | 30 | 8 | 14 | 21 | 23 |

never appear in code no. 162 or 163. Some certificates were worded in such a way that application of the coding rules demanded that another disease should take precedence over carcinoma of the lung in the allotted code number. For instance, in one certificate carcinoma of the bronchus appeared in part 1A but in part 1B an old tuberculous lesion of the lung appeared, to which disease the certificate was coded.

At this stage, inquiry from the Registrar-General was instituted to ascertain whether the coding of these 66 cases was similar to that adopted in Leeds. As the coding records for all years prior to 1954 had been destroyed, it was necessary to recode the certificates of all 66 cases and this was done by the method in use at the time and the method in current use. Two facts were ascertained: (1) in only 4 of 66 cases was there a difference of code number using the previous compared with the current method and (2) 7 cases were coded as carcinomas of the bronchus by the Registrar-General but had been coded by the Medical Officer of Health for Leeds in some other way. It thus seems probable that these 7 cases would form part of the 15 extra cases recorded by the Registrar-General (see p. 2).

TABLE IX.—*Estimate of the Degree of Accuracy of the Clinical Diagnosis of Cases in Two Hospitals*

| Category | Means of diagnosis | Male | Female | Total No. | Total Per cent |
|---|---|---|---|---|---|
| I | Clinical histology | 222 | 31 | 253 | |
| | Confirmed by autopsy | 156 | 19 | 175 | |
| | Diagnosed by autopsy | 88 | 17 | 105 | |
| | Total | 466 | 67 | 533 | 60·0 |
| II | Ancillary methods without histology | 301 | 44 | 345 | 38·8 |
| III | Physical examination and history | 9 | 2 | 11 | 1·2 |
| | Total | 776 | 113 | 889 | |

*Estimate of the degree of validity of diagnosis of the clinical cases*

In Table IX the clinical cases are classified according to international agreement. Omitting the 11 cases of which the notes were lost, it is seen that in 533 of 889 cases (60 per cent) a histological or autopsy diagnosis was made, in 345 (39 per cent) the diagnosis was made by ancillary methods and in only 11 (1·4 per cent) was it made by physical examination and history only. If 267 cases in class II, where the consultant gave the opinion that all the evidence (short of histological examination) was greatly in favour of a diagnosis of cancer of the bronchus, are added to class I, then a firm diagnosis was obtained in 92 per cent of cases.

### DISCUSSION

If death certificates are to be used as a basis for the analysis of data relating to the properties of any disease, the clinical basis on which they are founded must be as accurate as possible. It was thought that in a provincial town such as Leeds, where special facilities exist for the diagnosis and treatment of intrathoracic cancer, it should be possible to trace the clinical records of those patients certified as having died of this disease and to form an estimate of the degree of validity of the clinical diagnosis in relation to death certification. This was done by a doctor experienced in tracing and scrutinising clinical notes and we consider that this is a very necessary part of the investigation, as it greatly encourages the co-operation of hospital records officers, departmental secretaries, general practitioners and others. The knowledge and experience of a doctor are also required in order to interpret the notes. Search was continued until no other possible sources of information were left, with the result that only 37 of 1036 death certificates (3·6 per cent) were not traced (Table I).

Omitting these 37 certificates about which no clinical data could be obtained, it was found that the clinical diagnosis or autopsy record tallied with that recorded on the certificate in 964 of 999 cases, while in only 35 cases was the clinical diagnosis of another disease made in life and yet the certificate recorded death from carcinoma of the lung (Table I). The possible reasons for these discrepancies are discussed on p. 3, while the type of clinical diagnosis is shown in Table II. It is not difficult to understand why metastatic lung cancer (accounting for 23 cases) should occasionally be recorded as primary lung cancer, but it might be expected that non-cancerous conditions should not appear in this way.

In regard to the accuracy of the clinical diagnosis of cancer of the bronchus, the death certificates were traced back to the clinical notes in 953 cases (Table IV). In half of these, the diagnosis could be accepted as unequivocal (class I). In another 333 cases, the consultant in charge of the case was confident that the diagnosis was correct, leaving 143 cases (15 per cent) where the diagnosis was in considerable doubt. It is evident that where the clinical diagnosis was by no means certain (Table IX, class II) autopsy confirmed the condition in a large proportion of cases (Table V).

These findings are similar to those reported by McKenzie (1956) who used a different method of sampling. A questionary was sent from the General Register Office to the certifying medical practitioner in respect of every second death ascribed to cancer of the lung or bronchus in January, 1955. From 770 inquiries dispatched 654 replies were received. The validity of diagnosis was classified in much the same way as we have done and it was found that the standard of diagnostic technique was high: in only 3 per cent had no confirmatory procedure been adopted. Of a total of 634 certificates in only 18 cases was the certification of carcinoma of the lung not supported. In 8 of these the lung was found not to be carcinomatous and in the remaining 10 the lung condition was secondary to a primary elsewhere. It is interesting to note that in the 8 cases wrongly diagnosed as carcinoma the diagnosis was corrected in 4 by a post mortem examination made subsequent to the issue of the death certificate, and in the remaining 4, where necropsy apparently confirmed the diagnosis, subsequent histopathology disproved the macroscopic findings. The system obtaining in most hospitals, whereby the death certificate must be completed by the resident doctor before the post mortem examination is performed, leads in this way to a failure to amend the death certificate when the findings of the necropsy are known. It is always possible to overcome this difficulty if the resident doctor will initial Box B on the back of the certificate.

In addition to estimating the accuracy of the clincial data upon which the death certificates were based, an analysis was made of the mode of death certification of the cases diagnosed at the two main hospitals. Omitting the 20 patients known to be still alive, 813 of 879 cases diagnosed in the hospitals had death certificates which recorded the clinical diagnosis (8 of these were certified in another borough) and there were 66 in which cancer of the lung did not appear on the death certificate (7·5 per cent), or where the disease was not assigned to the coding carcinoma of the bronchus. In regard to the cases certified correctly, the clinical diagnosis was unequivocal in 60 per cent and highly probable in another 39 per cent (Table

IX). In the 66 cases certified as dying from other causes, the discrepancy was due either to the method of wording of the certificate or to difficulties in coding (see p. 8). Some of these discrepancies could have been avoided if the rules for certification and coding had been perfectly applied. But death certification does not and cannot be expected to give an exact picture of morbidity, as a few cases of cancer of the bronchus will be cured and others will die of independent causes, such as accidents and acute diseases.

An analysis of the clinical diagnosis in the 294 cases certified as dying of cancer of the lung and submitted to autopsy (Table V) is interesting. Where the autopsy was confirmatory of the clinical diagnosis, by far the largest group (76 per cent) had been diagnosed by ancillary methods only. In many of these cases there would either not be time to make a firm diagnosis or the consultant would consider that the evidence was strong, even in the absence of histological examination. Of the autopsies which revealed the condition for the first time, in one half another condition, either malignant or non-malignant, had been diagnosed and in the remaining half either no attempt at diagnosis had been made or the death was sudden. Of the 27 cases in which autopsy did not reveal cancer of the lung, but the death certificate recorded the condition, the diagnosis had been considered to be well established in 9 but in 17 there was no written clinical evidence for it and there was a real error in certification. This represents a false positive error in the death certificates of 9 per cent as revealed by autopsy, and this is largely contributed to by the 17 cases for which there was no written clinical evidence that the disease had ever been diagnosed in life. When the autopsy records of all the clinical cases in the two hospitals are analysed by Willis's method (Table IV) false positive diagnoses amount to 8 per cent.

The conclusion is drawn from all the above facts that the positive error in certification of cancer of the lung in Leeds is small (3·5 per cent). In fact, a larger number of cases which are diagnosed clinically fail to appear among the death certificates (7·5 per cent). The reasons for the errors are partly due to ambiguity of wording of the certificates, partly to mis-diagnosis and partly to the intervention of other causes of death.

It had been thought previously (Bonser and Thomas, 1955) that the discrepancy in certification was greater in females than in males and it was suggested that it was possible that more female deaths from metastatic lung cancer were recorded as primary lung cancer than were male deaths from this cause. No support could be obtained for this suggestion from this survey, 7 of 10 females and 16 of 25 males having been certified as dying from primary lung cancer when the disease was really metastatic (Table II). It was noted, however, that the female to male sex ratio was lower both for those cases incorrectly certified as lung cancer and for those positively diagnosed cases which were not certified as lung cancer. These effects tend to cancel one another out and there is no reason to suppose that the true sex ratio is materially different from that revealed by death certification.

### CONCLUSIONS

1. 1036 death certificates recorded in Leeds city in the years 1950–54 and coded as cancer of the trachea, pleura, lungs or bronchi (*i.e.* code no. 162 and 163) were scrutinised. The clinical records of all but 37 were traced. It was found that 3·4 per cent of the remaining 999 cases were incorrectly certified and that the remainder were certified on unequivocal evidence (50 per cent), probable evidence (45 per cent), and doubtful evidence (5 per cent).

2. The clinical notes of 879 similar cases recorded in the admission index of the two main hospitals in Leeds from 1950–54 were compared with their death certificates. 92·5 per cent were correctly certified at death and in 7·5 per cent the presence of the disease did not appear in the final coding by the Statistics department of the Medical Officer of Health for Leeds.

The reasons for the failure of cancer of the lung to appear in the coding might be : (*a*) the position of the words " cancer of the lung " on the certificate ; (*b*) failure to record carcinoma of the lung on the certificate at all ; or (*c*) the fact that the cancer of the lung was an incidental finding not causing death. More cases were diagnosed clinically and not certified at death, than were certified at death and not diagnosed clinically.

3. Analysis of the cases subjected to autopsy supported the finding that false positive certification was infrequent.

4. No sex difference in accuracy of certification was established.

We wish to record our thanks to Professor J. S. Young, University of Aberdeen, for the original suggestion that this investigation should be made, and to Dr. R. Doll for help and advice. We are also indebted to the Consultants in charge of patients in the hospitals in Leeds and elsewhere, to the hospitals Records Officers (in particular to Mr. Teale of the Leeds General Infirmary), and to secretaries of departments for help in searching for records. We owe a special debt to the Chief Statistical Clerk of the Statistics Department of the Medical Officer of Health, Leeds, to the Chairman and Secretary of the Leeds Executive Council and to many general practitioners for their constant help and willing co-operation. Acknowledgement is due to the staff of the Medical Statistics Department of the Registrar-General, Somerset House, for their help in the special search.

### REFERENCES

BONSER, G. M. AND THOMAS, G. M.—(1955) *Schweiz. Z. Path.*, **18**, 885.

McKENZIE, A.—(1956) *Brit. med. J.*, ii, 204.

Manual of the International Statistical Classification of Diseases, Injuries and Causes of Death. 6th Revision, Adopted 1948. Vol. I.

Symposium on the endemiology of cancer of the lung.—(1953) *Acta Un. int. Cancr.*, **9**, 440.

WILLIS, R. A.—(1948) 'Pathology of Tumours.' London : (Butterworth and Co. Ltd.) p. 69.

### ADDENDUM

Since the above communication went to press, an interesting comparison of diagnosis before and after post mortem has been made by the Registrar-General, who arranged for death certificates to be completed by a clinician immediately after death and by a pathologist immediately after post mortem in 1,404 cases in 10 hospitals in England. These represented 81·2 per cent of all deaths in these hospitals for the period under review, There was agreement in 51 per cent of cases, while in another 28 per cent the disagreement was one of opinion rather than of fact. Cancer of the lung was notably under-diagnosed by clinicians, the amount of error being greatest over the age of 65 years.

*Reference.*

Registrar-General's Statistical Review of England and Wales, 1956. Part III. Commentary, p. 182.